# Adjuvant crizotinib in high-risk uveal melanoma following definitive therapy

**DOI:** 10.3389/fonc.2022.976837

**Published:** 2022-08-29

**Authors:** Shaheer Khan, Jose Lutzky, Alexander N. Shoushtari, Joanne Jeter, Brian Marr, Thomas E. Olencki, Colleen M. Cebulla, Mohamed Abdel-Rahman, J. William Harbour, Naomi Sender, Alexandra Nesson, Shahnaz Singh-Kandah, Susana Hernandez, Jeanelle King, Manpreet S. Katari, Lyssa Dimapanat, Stephanie Izard, Grazia Ambrosini, Oliver Surriga, Alex J. Rai, Codruta Chiuzan, Gary K. Schwartz, Richard D. Carvajal

**Affiliations:** ^1^ Herbert Irving Comprehensive Cancer Center, Columbia University, New York, NY, United States; ^2^ Sylvester Comprehensive Cancer Center, University of Miami, Miami, FL, United States; ^3^ Melanoma and Immunotherapeutics Service, Department of Medicine, Memorial Sloan Kettering Cancer Center, New York, NY, United States; ^4^ The James Comprehensive Cancer Center, The Ohio State University, Columbus, OH, United States; ^5^ Department of Ophthalmology and Visual Sciences, Havener Eye Institute, The Ohio State University, Columbus, OH, United States; ^6^ Department of Melanoma Medical Oncology, University of Texas Southwestern Medical Center, Dallas, TX, United States; ^7^ Center for Genomics and Systems Biology, New York University, New York, NY, United States; ^8^ Feinstein Institutes for Medical Research, Northwell Health, New York, NY, United States

**Keywords:** uveal melanoma, high-risk, adjuvant therapy, crizotinib, extracellular vesicles

## Abstract

**Introduction:**

Approximately 40% of patients with uveal melanoma (UM) will develop metastatic disease. Tumors measuring at least 12mm in basal diameter with a class 2 signature, as defined by a widely used gene expression-profiling test, are associated with significantly higher risk of metastasis, with a median time to recurrence of 32 months. No therapy has been shown to reduce this risk.

**Materials and Methods:**

This was a single-arm, multicenter study in patients with high-risk UM who received definitive treatment of primary disease and had no evidence of metastasis. Patients were consecutively enrolled to receive 12 four-week cycles of adjuvant crizotinib at a starting dose of 250mg twice daily and were subsequently monitored for 36 months. The primary outcome of this study was to assess recurrence-free survival (RFS) of patients with high-risk UM who received adjuvant crizotinib.

**Results:**

34 patients enrolled and received at least one dose of crizotinib. Two patients were unevaluable due to early withdrawal and loss to follow-up, leaving 32 patients evaluable for efficacy. Eight patients (25%) did not complete the planned 48-week course of treatment due to disease recurrence (n=5) or toxicity (n=3). All patients experienced at least one adverse event (AE), with 11/34 (32%) experiencing a Common Terminology Criteria for Adverse Events (CTCAE) grade 3 or 4 AE. After a median duration of follow up of 47.1 months, 21 patients developed distant recurrent disease. The median RFS was 34.9 months (95% CI (Confidence Interval), 23-55 months), with a 32-month recurrence rate of 50% (95% CI, 33-67%). Analysis of protein contents from peripheral blood extracellular vesicles in a subset of patient samples from baseline, on-treatment, and off-treatment, revealed a change in protein content associated with crizotinib exposure, however without a clear association with disease outcome.

**Conclusions:**

The use of adjuvant crizotinib in patients with high-risk UM did not result in improved RFS when compared to historical controls. Analysis of blood extracellular vesicles revealed changes in protein content associated with treatment, raising the possibility of future use as a biomarker. Further investigation of adjuvant treatment options are necessary for this challenging disease.

## Introduction

Uveal melanoma (UM) arises from melanocytes within the uveal tract and is the most common primary intraocular malignancy in adults (1). While most patients are free of measurable disease after definitive therapy to the primary tumor, approximately 40% of patients will develop metastatic disease within 15 years of diagnosis (2, 3). The most common initial site of metastatic disease is the liver; however, other common sites include the lungs, soft tissue, and bone (4). Several clinical features, including tumor size, ciliary body involvement, and extraocular extension, are associated with an increased risk of metastatic disease (5). In addition to cytogenetic abnormalities, such as monosomy 3, which have been strongly associated with clinical outcome, a prospectively validated 15-gene expression profile (GEP) is also frequently used for risk-stratification (6, 7). This assay categorizes patients as Class 1a (lower risk), Class 1b (intermediate risk) and Class 2 (higher risk), with 5-year metastatic risk of 2%, 21% and 72%, respectively, albeit strongly associated with tumor size (6, 8). Several analyses have demonstrated that patients with Class 2 GEP and largest basal diameter ≥12mm are at highest risk of metastasis. One large series found that metastasis-free survival at 5 years in UM tumors with Class 2 GEP and LBD (Largest Base Diameter) ≥12mm was only 25% (9). Comparable results were found in another large study with two independent cohorts (10).

To date, there is no approved systemic therapy that has been shown to reduce the risk of metastasis or improve recurrence-free survival (RFS) or overall survival (OS) in the adjuvant setting (11–15). Recent results from a randomized phase II trial of adjuvant sunitinib or valproic acid in patients with high-risk UM showed a tendency toward improved outcomes with sunitinib compared to historical controls. These data are being further assessed with longer follow up and an additional cohort (16). Earlier this year, tebentafusp became the first FDA approved therapy for the treatment of metastatic uveal melanoma, however it is restricted to patients who are HLA-A*02:01–positive. Given the poor prognosis and limited treatment options associated with metastatic disease, the development of effective adjuvant therapies is critical to the management of patients at high risk of recurrence.

cMET, or hepatocyte growth factor (HGF) receptor, is a protein encoded by the MET gene and normally expressed in cells of epithelial origin. HGF, the ligand for cMET, is typically produced in mesenchymal cells including hepatic stellate cells (17). Although activating mutations or genetic amplifications of cMET are not a characteristic finding in UM (18), high expression of cMET has been observed in 60%-86% of UM cases (18). cMET overexpression is associated with greater cell migration capacity and inferior clinical outcomes (19, 20). In a series of 60 patients with resected UM, higher levels of cMET expression were associated with a significantly higher risk of death from metastatic disease (21). In addition, there is data to suggest that soluble levels of cMET can be used a as a biomarker of metastatic disease (22).

Crizotinib, a selective small-molecule tyrosine kinase inhibitor (TKI) of cMET, anaplastic lymphoma kinase (ALK), and reactive oxygen species (ROS1) is approved for ALK or ROS1-positive metastatic non-small cell lung cancer (23, 24). In prior work from our group using uveal melanoma cell lines transfected with cMET siRNA, downregulation of cMET resulted in decreased cell proliferation and migration (25, 26). Orthotopic xenograft mouse models transplanted with UM cells treated with crizotinib demonstrated suppression of metastatic spread with treatment compared to control mice (25). Based on this biological relevance of the cMET axis in UM as well as our preclinical data supporting the anti-migratory and anti-tumor activity of MET inhibition, we performed a phase II clinical trial to investigate our hypothesis that MET inhibition with crizotinib would prevent the establishment and development of metastases in patients with high-risk primary UM.

## Materials and methods

**Figure 1:**
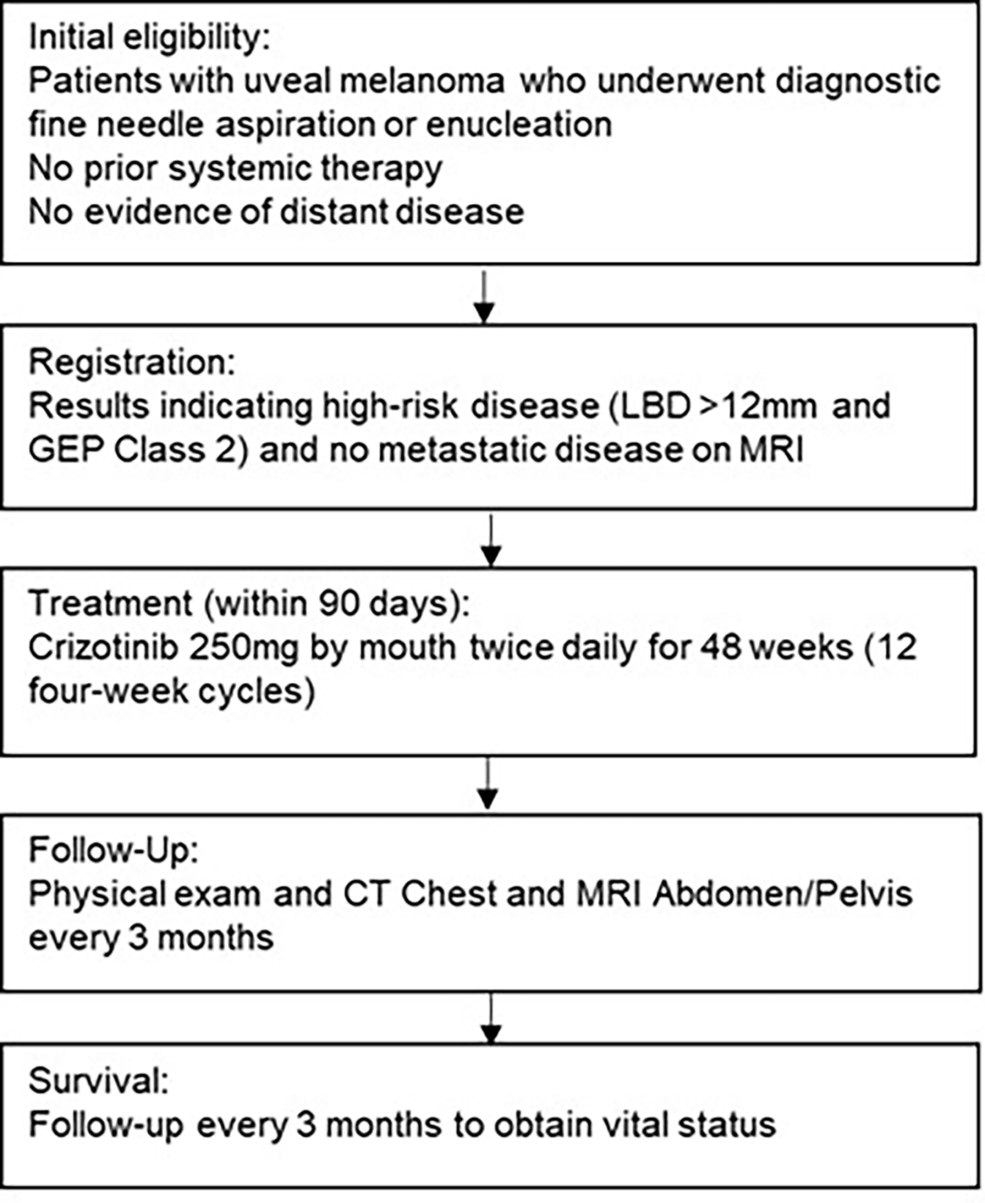
Trial Design.

Between March 2015 and January 2018, 34 patients with high-risk UM (LBD >12mm, GEP Class 2) from 4 academic medical centers were enrolled. Eligible patients received crizotinib at a dose of 250mg twice daily for 12 four-week cycles (**Figure 1**). The primary efficacy endpoint was recurrence-free survival (RFS) rate at 32 months. This outcome was based on data from a large retrospective observational study which found a median PFS of 32 months in patients with Class 2 GEP and basal diameter >12mm (24). Secondary endpoints included overall survival (OS), disease-specific survival, and toxicity. The protocol was approved by the institutional review board at each respective institution and conducted under the principles of the International Council of Harmonization and Good Clinical Practice. Drug and funding for this investigator-sponsored research study was provided by Pfizer, Inc (New York, NY, USA) and was registered with www.clinicaltrials.gov as NCT02223819 . All patients provided written informed consent prior to enrollment. The sponsor had no role in data collection, analysis, or interpretation, or in writing of this report.

### Patient selection

Patients 18 years of age or greater with UM measuring at least 12mm in LBD and Class 2 as determined by DecisionDx-UM GEP (Castle Biosciences, Friendswood, TX, USA) were eligible for this study. Diagnosis and size of melanoma was confirmed based on clinical assessment (e.g., using ultrasound, photography, and ophthalmoscopy) prior to enucleation or radiation therapy. Patients must have had no evidence of disease on baseline CT (Computed Tomography) scan of the chest and MRI (Magnetic Resonance Imaging) of the abdomen and pelvis with contrast and a performance status (ECOG) of 0 or 1. Additional inclusion criteria comprised the ability to provide consent, normal organ and marrow function, and life expectancy greater than 3 months. Patients had to enroll within 90 days of completing primary therapy. Exclusion criteria included prior crizotinib use, other investigational drugs within 4 weeks or 5 half-lives, another malignancy within 3 years, prolonged QTc interval, known Human Immunodeficiency Virus (HIV), Hepatitis B Virus (HBV), or Hepatitis C Virus (HCV) infection (except for chronic or cleared HBV and HCV infection) or uncontrolled other illness.

### Patient monitoring for efficacy and adverse effects

Dose-limiting toxicities (DLTs) were graded according to the National Cancer Institute Common Terminology Criteria for Adverse Events (CTCAE) version 4.0. Patients were monitored with complete blood counts with a differential, comprehensive metabolic panel, and physical exam at baseline and every 4 weeks during the study. Surveillance imaging including CT Chest and liver MRI were performed every 12 weeks during and after completion of therapy. Dosing was held and modified for grade 3 or 4 toxicities.

### Correlative analysis

Peripheral blood samples were collected at baseline and every 4-12 weeks for correlative analysis. Isolated RNA was preserved at the time of GEP analysis if sufficient material were available. If consented to by the subject and deemed safe, tumor samples at the time of disease recurrence were also collected for subsequent analysis. Extracellular vesicles were isolated from serum and plasma samples using EVtrap. Quantitative mass spectrometry was used for proteomic characterization (27, 28).

### Statistical analysis

The primary objective of this phase II, single-stage trial was to determine the 32 months RFS rate in patients with uveal melanoma. RFS rate was defined as the percentage of patients who do not experience any new tumor growth at any site on the body distant from the primary site or death from any cause from the time of primary therapy of the uveal melanoma (date of enucleation or day of removal of the radioactive plaque) to 32 months. The trial was powered to detect a target RFS of 75% at 32 months, versus a null hypothesis of 50% based on historical data. The planned enrollment to detect this difference was 30 patients, granting 90% power with a two-sided type I error of 0.05. At the end of the study, if at least 20 out of 30 patients were free of distant recurrence at 32 months, then adjuvant treatment crizotinib would be considered worthy of further study. Secondary endpoints included overall survival (OS), disease-specific survival (DSS), safety and tolerability of adjuvant treatment with crizotinib. OS was defined as the time from treatment start to date of death or last follow-up. Patients were censored at their last known date of contact. RFS and OS probabilities were estimated using Kaplan–Meier method; medians (95% CI) were reported as well as probabilities at 12, 24, 32, and 36 months.

Additionally, we leveraged patient information from an existing database collected by the Cooperative Ocular Oncology Group (COOG) to develop an external synthetic control for this trial treatment group. We employed propensity score methods (PSM) to generate matched pairs to provide an accurate estimate of the reduction in RFS rate due to treatment with adjuvant crizotinib in high-risk UM patients (largest basal diameter >12mm and GEP class 2).

Patients with available RFS outcomes were matched in a 2:1 fashion based on four factors including age, sex, base diameter, and initial treatment. We created a matched sample by matching trial and database subjects on the logit of the propensity score using a greedy, nearest-neighbor matching algorithm with and without calipers. For calipers we used widths equal to 0.1 and 0.2 of the standard deviation of the logit of the propensity score. Standardized differences were calculated to assess the balance diagnostics between the trial arm and the external control. The standardized differences were compared before and after matching with imbalance being defined as an absolute value greater than 0.10 (small effect size) (29). The matching option that used all the trial data (N=32) and generated the best comparability with the database was not based on calipers. Stratified log-rank tests were employed to compare the equality of the RFS and OS curves between the trial and synthetic controls matched arms.

## Results

### Patient demographics

A total of 34 patients with uveal melanoma were enrolled and received at least one dose of study drug (**Table 1**). The median age was 60 (range, 26-86) with a median ECOG performance status of 0 (range, 0-1) and 41% of patients were female. All patients were Class 2 by GEP, with a median LBD of 14.0mm (range 12-21mm). Most tumors were in the posterior choroid with 9% of tumors involving the ciliary body. For definitive control of the primary tumor, 10 patients (29%) underwent enucleation and 24 (71%) underwent definitive radiotherapy (proton beam therapy or brachytherapy).

**Table 1:** Baseline Characteristics.

Baseline Characteristics (n=34)	
**Age at diagnosis (median, range)**	60 years (26-86)
**Male** **Female**	20 (59%) 14 (41%)
**ECOG status (median, range)**	0 (0-1)
**Race** **White** **Hispanic** **Other/Unknown**	25 (74%) 2 (6%) 7 (20%)
**Largest basal diameter (median, range)**	14.0 mm (12-21mm)
**Ciliary body involvement** **Yes** **No**	3 (9%) 31 (91%)
**Primary treatment modality** **Radioactive plaque** **Surgery**	24 (71%) 10 (29%)

### Treatment

The average time between definitive therapy and initiation of adjuvant crizotinib was 63 days. The median number of treatment cycles was 12 (range: 1-12). A total of 14 patients had treatment held or reduced while on study due to toxicity (n=10) or disease recurrence (n=4). Among the patients who had to stop treatment due to toxicity, the reported causes were persistent liver enzyme elevation, gait disturbance, interstitial lung injury, and myocardial infarction. The mean relative dose intensity across the entire group was 84%, with 18 patients achieving a dose intensity of over 90%. Dose intensity was calculated per cycle (received dose/planned dose). Total planned dose was not used for patients who discontinued therapy due to toxicity or disease progression. Four patients did not complete the full 48-week course of treatment due to persistent toxicity despite dose modification.

### Efficacy

**Figure 2:**
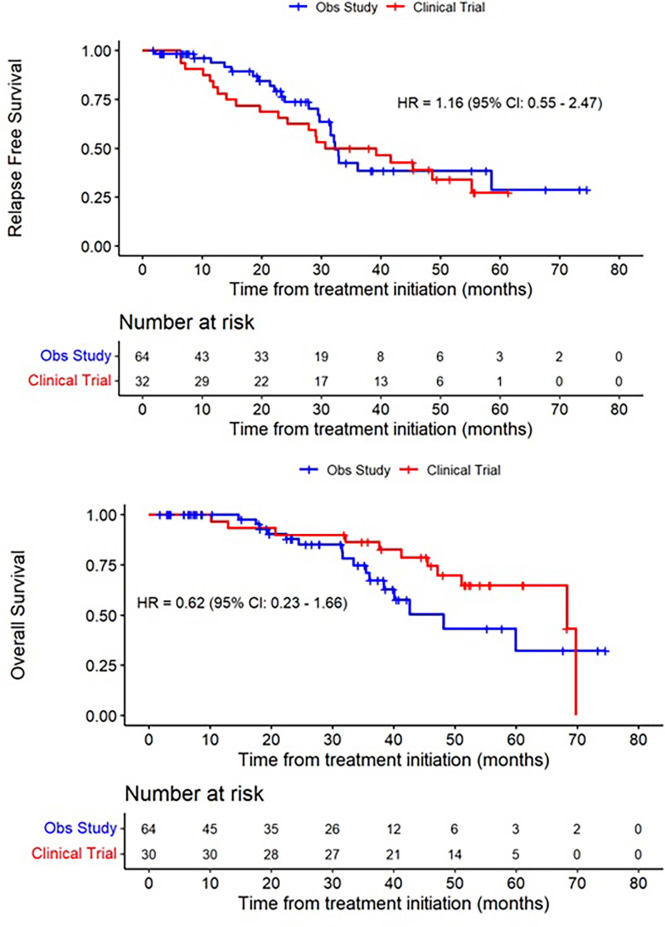
Recurrence-free Survival and Overall Survival of treated patients (red), compared to a synthetic control arm (blue).

Of the 34 patients who enrolled, 2 patients were unevaluable for assessment of the primary endpoint due to early withdrawal and loss to follow-up, leaving 32 patients evaluable for efficacy. Among these patients, 21 developed distant recurrence, with 16 developing recurrences within 32 months. Five patients did not complete the full 48-week course of treatment due to disease recurrence. Among patients who developed distant recurrence, the most common site was the liver or liver + other site (n=17, 81%), consistent with known patterns of spread in UM. Other sites of distant recurrence were lung, bone, and kidney (**Table 2**). After a median duration of follow up of 47.1 months, the median RFS in the study population was 34.9 months (95% CI 22.8-55.2), corresponding with a 32-month recurrence rate of 50% (95% CI, 23-67%); (**Figure 2** and **Table 3**). The median OS in the study population was 68.3 months (95% CI, 51.0-NA). A total of 11 deaths have been confirmed, all from disease progression. Two additional patients were lost to follow up after confirmed disease recurrence for whom survival status is not known.

**Table 2:** Site of Distant Recurrence.

Site of distant recurrence (%)	
**Liver only**	14 (67)
**Liver + other site**	3 (14)
**Lung**	2 (10)
**Kidney**	1 (5)
**Bone**	1 (5)

**Table 3:** Recurrence-free survival probabilities.

Timepoint	Clinical Trial RFS (95% CI)	Synthetic Control RFS (95% CI)
**12 months**	81.2% (67.7-94.8%)	93.9% (87.2-100%)
**24 months**	65.6% (49.2-82.1%)	73.8% (60.2-87.3%)
**32 months**	50.0% (32.7-67.3%)	56.6% (39.8-73.4%)
**36 months**	50.0% (32.7-67.3)	42.5% (25.1-59.9%)

Using propensity scoring as described above, we identified a matched population based on demographic factors including age, sex, base diameter, and primary treatment modality. In this matched population, the median RFS was 32.3 months (95% CI 29.7-58.5), and median OS was 48.1 months (95% CI 37.4-NA).

### Safety

A summary of adverse events is provided in **Tables 4**, **5**. All patients experienced at least one adverse event related to therapy (AE), with 11/34 (32%) experiencing a grade 3 or 4 AE. The most common grade 3 or 4 AE was transaminase elevation. One patient experienced a myocardial infarction while on therapy. Despite the overall rate of AE, most patients completed study therapy with limited dose interruptions or dose reductions. The mean relative dose intensity was 84% in the total population, with over half of patients achieving a relative dose intensity of over 90%.

**Table 4:** All Cause Adverse Events >10%.

Adverse Events During Treatment			
Term	Any Grade (%)	Grade 3 (%)	Grade 4 (%)
**Adverse Event**	34 (100)	11 (32)	1 (3)
Nausea	20 (59)	0	0
Diarrhea	17 (50)	2 (6)	0
Fatigue	17 (50)	1 (3)	0
Edema	17 (50)	0	0
Sinus Bradycardia	15 (44)	0	0
Constipation	12 (35)	0	0
Dyspepsia/GERD	12 (35)	0	0
Dizziness	11 (32)	0	0
Dysgeusia	7 (21)	0	0
Floaters	7 (21)	0	0
Vomiting	7 (21)	0	0
Cough	6 (18)	0	0
Flashing lights	5 (15)	0	0
Headache	5 (15)	0	0
Hypertension	5 (15)	0	0
Blurred Vision	4 (12)	0	0
Anorexia	3 (9)	0	0
Paresthesia	3 (9)	0	0
Rash	3 (9)	0	0
Dysphagia	3 (9)	0	0
QTC prolongation	2 (6)	1 (3)	0
VTE (Venous Thromboembolism)	2 (6)	1 (3)	0
Myocardial Infarction	0	1 (3)	0
Syncope	0	1 (3)	0
**Laboratory Parameters**			
Transaminase elevation	16 (47)	7 (20%)	0
Leukopenia	10 (29)	0	0
Hypoalbuminemia	9 (26)	0	0
Elevated Creatinine	8 (24)	0	0
LDH elevation	8 (24)	0	0
Hypocalcemia	7 (21)	0	0
Anemia	7 (21)	0	0
Hyperkalemia	6 (18)	0	0
Hyperglycemia	6 (18)	0	0
Hypophosphatemia	3 (9)	0	0
Thrombocytopenia	3 (9)	0	0

**Table 5:** Most Common Treatment-Related Adverse Events.

	Any Grade	Grade 1-2	Grade 3	Grade 4
**All Events **	34 (100%)	23 (68%)	11 (32%)	0
**Most common adverse events**				
Nausea	18 (53)	18 (53)	0	0
Transaminase elevation	16 (47)	9 (26)	7 (21)	0
Diarrhea	17 (50)	15 (43)	2 (6)	0
Fatigue	17 (50)	16 (47)	1 (3)	0
Sinus bradycardia	15 (43)	15 (43)	0	0
**Dose Modification** 200mg twice daily 250mg once daily Discontinuation	9 (26) 2 (6%) 3 (9%) 4 (12%)	
**AE’s leading to discontinuation **	LFT elevation Gait disturbance Interstitial lung disease MI			

### Correlative analysis

#### Extracellular vesicle proteome analysis

**Figure 3:**
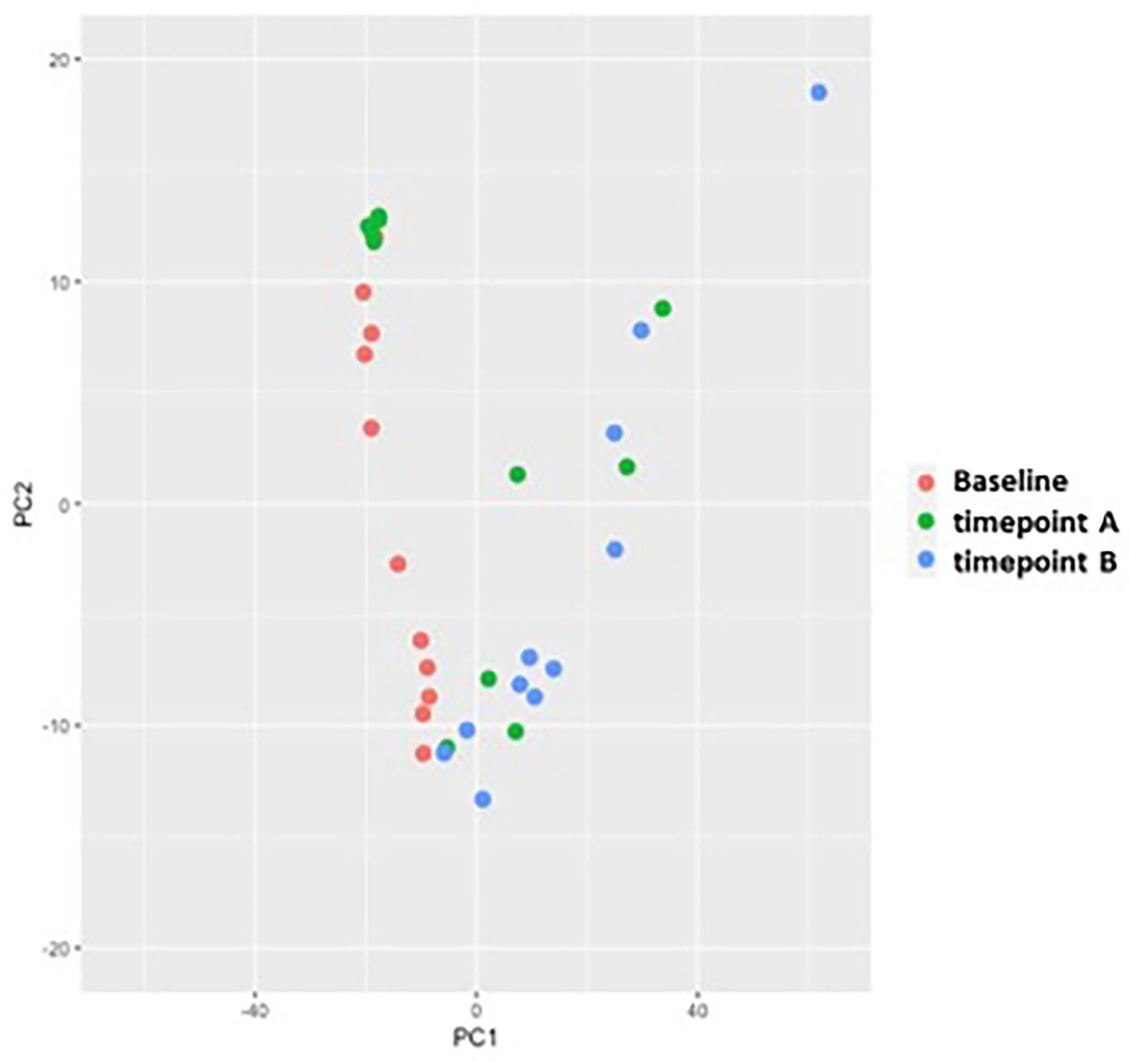
Principal component analysis (PCA) of protein profiles by timepoint showing clustering of baseline (red) and (blue) timepoint B samples with distribution of (green) timepoint A across both clusters.

**Figure 4:**
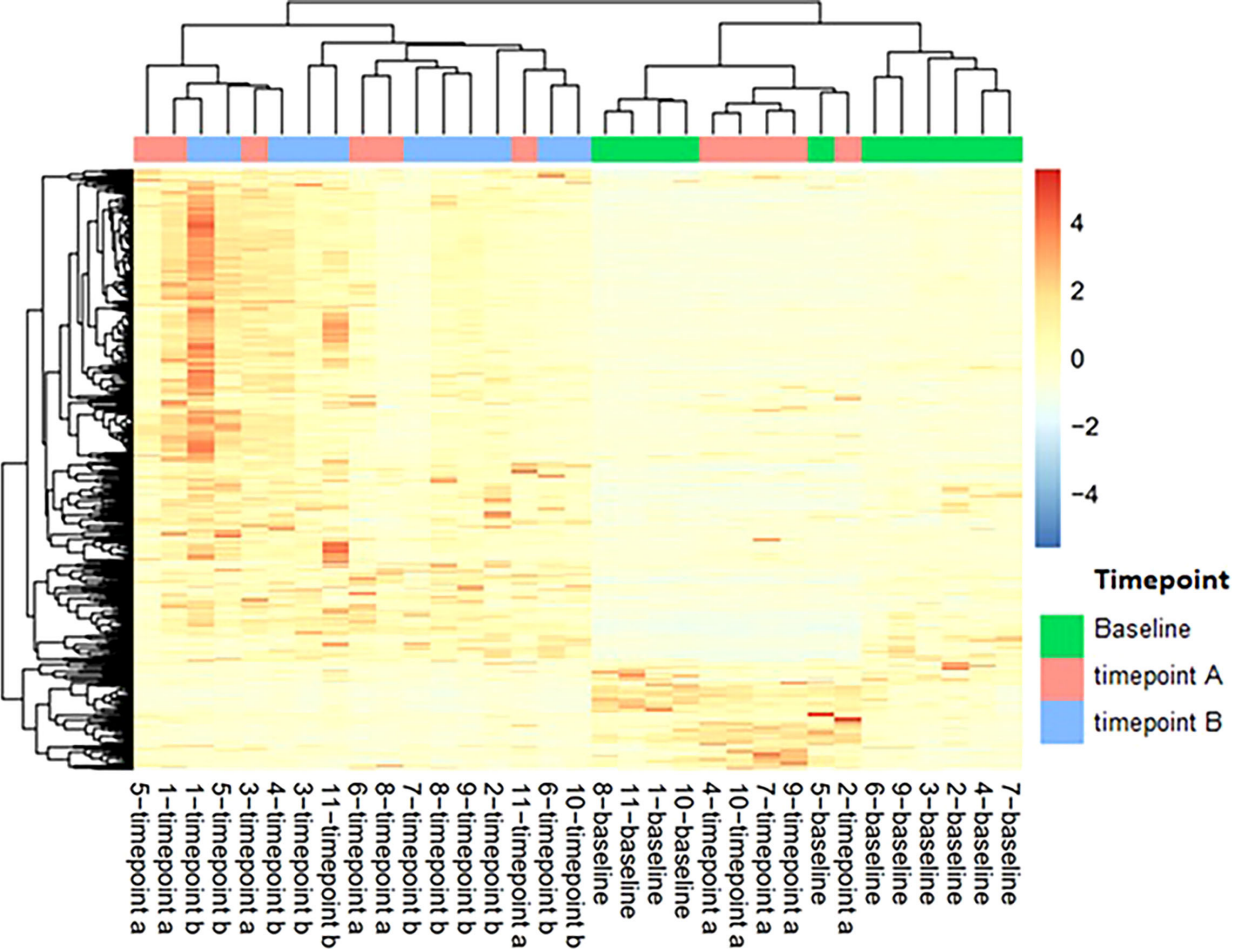
Heatmap of extracellular vesicle protein expression with hierarchical clustering of signatures by patient and timepoint. Timepoint A corresponds to week 12 on-treatment. Timepoint B corresponds to weeks 60-84 off-treatment. Patients 4, 5, 7, and 10 developed recurrence.

Peripheral blood samples from 11 patients were assessed for extracellular vesicle protein content at 3 timepoints for 10 patients and 2 timepoints for 1 patient; baseline, on-treatment (week 12), and off-treatment (week 60-84) (**Table 6**). Over 2000 proteins were identified from each of the plasma samples. PCA analysis was used to assess the relationship of the overall protein profiles of all 32 samples (**Figure 3**). Hierarchical clustering using unsupervised analysis revealed a signature that separated the dataset into two major clusters, delineated by the baseline and off-treatment samples, with the on-treatment samples distributed almost evenly between the two. When stratified by outcome, 3 of 4 patients who developed recurrence failed to show a change in protein signature at week 12 (timepoint A), suggesting a potential association with a lack of treatment effect (**Figure 4**).

**Table 6:** Samples analyzed for extracellular vesicle protein content.

Subject	Timepoint #1	Timepoint #2	Timepoint #3	Disease Recurrence?	Site of Metastasis
**1**	Baseline	Week 12	Week 72	Y	Lung
**2**	Baseline	Week 12	Week 72	Y	Lung
**3**	Baseline	Week 12	Week 72	N	
**4**	Baseline	Week 12	Week 72	N	
**5**	Baseline	Week 12	Week 84	Y	Liver
**6**	Baseline	Week 12	Week 84	N	
**7**	Baseline	Week 12	Week 72	Y	Liver/Lung
**8**	Baseline	Week 12	Week 60	N	
**9**	Baseline	Week 12	Week 60	N	
**10**	Baseline	Week 12	Week 72	N	
**11**	Baseline	Week 12	XX	N	

## Discussion

In this prospective, single arm, phase II study investigating the efficacy and tolerability of adjuvant crizotinib in patients with high-risk uveal melanoma, we observed a 32-month distant RFS rate of 50% which did not meet our pre-specified endpoint for efficacy of 75%. Given the promising efficacy of crizotinib in the preclinical setting, the failure of crizotinib to prevent or delay distant recurrence in patients with high-risk UM is disappointing and warrants further research to understand potential mechanisms for the lack of effect.

As part of this clinical trial, peripheral blood was collected at baseline and at regular intervals on and after completion of treatment to assess for changes in extracellular vesicle protein content from a subset of patients. Among these 11 patients, which included 4 patients in whom distant recurrence has developed, we found a change in the protein content in on-treatment and off-treatment samples, compared to baseline. Although the extracellular vesicles that were analyzed cannot be isolated to those secreted by uveal melanoma cells, the demonstration of a change in protein cargo suggests that extracellular vesicle protein expression has the potential for use as a tool to assess treatment effect. In our study, we identified a potential relationship between a change in protein expression and development of distant recurrence as 3 of 4 patients with confirmed distant recurrence did not have a change in protein content after 12 weeks of treatment. This finding, although limited by many potential confounding variables, does correlate with previous data suggesting a role of extracellular vesicle cargo in priming of the metastatic niche and may warrant further investigation as a potential biomarker (31).

Among the potential options to explain the discordant findings from preclinical data and our trial results, the limitations of available preclinical models of this disease may play a significant role. The animal model used in preclinical assessments used to justify this study were severe combined immunodeficient (SCID) mice bearing subcutaneously injected UM cells. Although these models have been instrumental in elucidating critical signaling pathways and testing new therapeutic strategies for this disease, it is possible the immunodeficient status of the model may have impacted the effect of crizotinib in preclinical testing. There is also increased awareness that uveal melanoma cell lines may harbor differences from their parental tumor due to changes that can result from culturing *in-vitro* (31, 32). One mechanism that has been proposed to potentially avoid these issues is by utilization of patient-derived xenografts from freshly acquired tumor specimens, including hepatic tumors. Although these models would still require implantation in immunodeficient mice, they may better recapitulate the behavior of UM in humans (33, 34).

An additional potential hypothesis to explain the lack of benefit in this study is that toxicity related to study therapy may have resulted in decreased compliance and sub-therapeutic levels of crizotinib. However, despite discontinuation or dose reduction in 14 of 34 patients, the mean relative dose intensity was 84% across all patients, with a significant fraction of patients achieving a dose intensity over 90%. In addition, among the 24 patients who completed the full course of crizotinib, the median RFS was only 29 months. These findings suggest that compliance with the prescribed regimen does not explain the lack of efficacy.

As has been demonstrated in several other clinical trials studying targeted therapies in metastatic UM, it is also possible that inhibition of a single therapeutic target is not sufficient to inhibit the growth of UM in humans. Previous studies have tested inhibition of several targets/pathways in the metastatic setting and have failed to demonstrate significant benefit, including VEGF (Vascular Endothelial Growth Factor), MEK, and PKC inhibitors (35–37). This has led to more recent trials combining multiple targets in an effort to improve response. Preclinical data have demonstrated that cMET may mediate resistance to MEK inhibition, which raises the possibility that combined inhibition of cMET with other targets downstream of GNAQ/GNA11 such as MEK, PKC, or PI3K (phosphoinositide 3 kinase), may offer more substantive benefit than inhibition of cMET alone (38). Crizotinib is currently being investigated in a phase I/II trial in combination with the PKC inhibitor darovasertib (NCT03947385).

One potential weakness in interpreting the results of small single arm trials such as this one is the lack of a randomized control arm. Randomized clinical trials are the gold standard in therapeutic development; however, in rare diseases such as UM, adequate enrollment in randomized clinical trials can be difficult due to the limited number of cases and the hesitance of patients to be placed on a placebo arm. Similar to other modifications such as randomized discontinuation trials, using a synthetic control arm comprised of matched historical controls may provide an alternative mechanism to enable trial development and enrollment. This study used a synthetic control arm deriving from a cohort of patients with high-risk UM (largest basal diameter >12mm and GEP class 2) with a matched population based on factors including age, sex, base diameter, and initial treatment. By using this study design, our trial demonstrated that there was also a lack of benefit not just compared to historical data, but also when compared to a matched cohort of patients.

## Conclusions

The need for effective treatment options to reduce the risk of distant metastatic disease in patients with high-risk uveal melanoma is urgent. In this novel phase II multicenter trial of adjuvant crizotinib in high-risk patients, the median RFS was 34.9 months, which was similar to historical outcomes as well as a matched synthetic control arm, and did not meet the pre-specified endpoint of a 32-month RFS rate of 75%. Crizotinib was associated with significant toxicities in 11 of 34 evaluable patients and required discontinuation in 4 patients. The mechanisms by which resistance to crizotinib occurred are unclear, however points to the limitations of current preclinical models in this disease and our understanding of the biological effects of targeted therapies in patients. Although this trial did not meet its pre-specified endpoint, it represents a significant effort to complete a multicenter prospective trial in a difficult to study patient population.

## Data availability statement

The raw data supporting the conclusions of this article will be made available by the authors, without undue reservation.

## Ethics statement

The studies involving human participants were reviewed and approved by the protocol was approved by the institutional review board at each respective institution. The patients/participants provided their written informed consent to participate in this study.

## Author contributions

Conceptualization, RDC, GA, OS, JL, and GKS; trial conduct, BM, NS, SS-K, SH, JK and AN; analysis, SK, ANS, JL, BM, CCe, MA-R, JWH, MSK, LD, SI, AJR, CCh, GKS, and RDC; writing—original draft preparation, SK and RDC; writing—review and editing, SK, JL, ANS, CCe, AJR, CCh, and RDC; supervision, RDC. All authors have read and agreed to the published version of the manuscript.

## Funding

Drug and funding for this investigator-sponsored research study was provided by Pfizer, Inc (New York, NY, USA). The sponsor had no role in data collection, analysis, interpretation, or in writing of this report.

## Conflict of interest

SK reports honoraria from Castle Biosciences. JL reports advisory/consulting fees from Regeneron, Sapience Therapeutics, Agenus, and research funding from Bristol Myers Squibb, Novartis, Agenus, Vyriad, Regeneron, Immunocore, Foghorn, Replimmune, InstilBio, Iovance, InflaRx, and Trisalus. ANS reports advisory/personal fees from Bristol Myers Squibb, Immunocore, Novartis, and research funding from Pfizer, Bristol Myers Squibb, Immunocore, Novartis, Targovax, Polaris, Checkmate Pharmaceuticals, Foghorn Therapeutics, Linneaus Therapeutics, and Prelude Therapeutics. GKS reports stock or other ownership interests in GenCirq, Bionaut Labs, and January Therapeutics; advisory/consulting/personal fees from Bionaut Labs, Ellipses Pharma,GenCirq, Epizyme, Array BioPharma, Apexigen, Oncogenuity, OnCusp Therapeutics, Concarlo, Shanghai Pharma, Astex Pharmaceuticals, January Therapeutics, Sellas Life Sciences, PureTech Health, and Killys Therapeutics; research funding from Astex Pharmaceuticals, Incyte, Calithera Biosciences, Lilly, Daiichi Sankyo, Fortress Biotech, Karyopharm Therapeutics, Oxford Biotherapeutics, TopAlliance Biosciences, Adaptimmune, Springworks Therapeutics, and TRACON Pharma. RDC reports consulting fees from Alkermes, Bristol Myers Squibb, Castle Biosciences, Delcath Systems, Eisai, Jiangsu Hengrui Pharmaceuticals, Ideaya Biosciences, Immunocore, InxMed, Iovance, Merck, Novartis, OncoSec, Pierre Fabre, PureTech Health, Regeneron, Sanofi Genzyme, Sorrento Therapeutics, Trisalus and advisory board fees from Aura Biosciences, Chimeron, and Rgenix.

The remaining authors declare that the research was conducted in the absence of any commercial or financial relationships that could be constructed as a potential conflict of interest.

## Publisher’s note

All claims expressed in this article are solely those of the authors and do not necessarily represent those of their affiliated organizations, or those of the publisher, the editors and the reviewers. Any product that may be evaluated in this article, or claim that may be made by its manufacturer, is not guaranteed or endorsed by the publisher.
